# Potent response of QS-21 as a vaccine adjuvant in the skin when delivered with the Nanopatch, resulted in adjuvant dose sparing

**DOI:** 10.1038/srep29368

**Published:** 2016-07-11

**Authors:** Hwee-Ing Ng, Germain J. P. Fernando, Alexandra C. I. Depelsenaire, Mark A. F. Kendall

**Affiliations:** 1 The University of Queensland, Delivery of Drugs and Genes Group (D^2^G^2^), Australian Institute for Bioengineering & Nanotechnology, Brisbane, Queensland 4072, Australia; 2 ARC Centre of Excellence in Convergent Bio-Nano Science and Technology, The University of Queensland, Brisbane, Queensland, Australia; 3 The University of Queensland, Faculty of Medicine and Biomedical Sciences, Royal Brisbane and Women’s Hospital, Herston, Queensland 4006, Australia

## Abstract

Adjuvants play a key role in boosting immunogenicity of vaccines, particularly for subunit protein vaccines. In this study we investigated the induction of antibody response against trivalent influenza subunit protein antigen and a saponin adjuvant, QS-21. Clinical trials of QS-21 have demonstrated the safety but, also a need of high dose for optimal immunity, which could possibly reduce patient acceptability. Here, we proposed the use of a skin delivery technology – the Nanopatch – to reduce both adjuvant and antigen dose but also retain its immune stimulating effects when compared to the conventional needle and syringe intramuscular (IM) delivery. We have demonstrated that Nanopatch delivery to skin requires only 1/100^th^ of the IM antigen dose to induce equivalent humoral response. QS-21 enhanced humoral response in both skin and muscle route. Additionally, Nanopatch has demonstrated 30-fold adjuvant QS-21 dose sparing while retaining immune stimulating effects compared to IM. QS-21 induced localised, controlled cell death in the skin, suggesting that the danger signals released from dead cells contributed to the enhanced immunogenicity. Taken together, these findings demonstrated the suitability of reduced dose of QS-21 and the antigen using the Nanopatch to enhance humoral responses, and the potential to increase patient acceptability of QS-21 adjuvant.

Adjuvants can be crucial components in vaccines. Adjuvants broaden the immune response, particularly for the poorly immunogenic subunit protein type antigens^1^. Subject to the adjuvant’s nature, immune responses can be enhanced and/or skewed towards a particular cellular/humoral response and various cell infiltrations^2^. In many instances, an adjuvant can induce responses adequate for protection with only a single vaccination, potentially reducing the cost of vaccinations and patient compliancy issues^3^.

A semi-purified saponin adjuvant, Quil-A (QA), is widely used in veterinary applications and has shown to induce strong humoral and cellular responses^4^,^5^. This is supported by our previous studies in mice, where we demonstrated the enhancement of antigen specific IgG and CD8^+^ T cell responses upon Nanopatch immunisations^6^,^7^. However, QA is considered unsuitable for human use due to its highly complex mixture nature and some components which could lead to toxicity and safety issues^8^,^9^. Therefore, an alternative to QA such as QS-21 (a highly purified component of QA) has been developed.

QS-21 is a promising adjuvant candidate for use in humans due to the ease of purification, its improved safety profile, and its ability to enhance cellular and humoral immunogenicity^8^,^9^,^10^. The mechanism of action of QS-21 was speculated to be similar to QA, forming complexes with cholesterol that intercalate into the cell membrane lipids^9^,^11^. This intercalation creates pores in the membrane to accelerate the uptake of a co-delivered antigen by the antigen presenting cells (APCs)^8^,^11^. A recent study has also indicated an inflammasome activation mechanism of QS-21^12^. Even though the specific mechanism of action of this adjuvant is unclear, several human clinical trials (e.g. Malaria and Herpes Zoster vaccine) included QS-21 as adjuvant due to its safety profile and the ability to enhance immunogenicity^13^,^14^.

Multiple clinical trials using QS-21 as adjuvant, demonstrated satisfactory safety profiles and enhanced immunogenicity in immunocompromised volunteers, namely the young (5 to 17 months)^13^,^15^ and the old (50 years and above)^14^. Memory responses have been observed 4 years post vaccination, in volunteers aged 22 to 45 years old to Hepatitis B vaccine with QS-21 as an adjuvant^16^. Malaria vaccine (with QS-21 as a component of the adjuvant) is currently under review for the regulatory application to European Medicines Agency to be licensed for human use^17^,^18^. These studies showed the safety and enhanced immunogenicity of QS-21 in IM-based vaccinations. Studies on other delivery routes such as skin delivery using QS-21 are limited, to our knowledge.

We have shown that our skin delivery device (Nanopatch) successfully generates potent immune responses and dose sparing (compared to IM) with many antigens: including ovalbumin^7^,^19^, trivalent influenza subunit protein (Fluvax)^6^,^20^,^21^, live viral vector encoding malaria antigen vaccine^22^; and adjuvants such as QA and CpG ODN^7^, amongst others. The mouse version of the Nanopatch is a 4 by 4 mm microprojection array that consists of 110 μm long projections (about 21,000 per cm^2^), designed to deliver antigen into the vicinity of APCs in the viable epidermis and dermis of the skin^6^.

Skin-based vaccine delivery routes such as intradermal (ID) injections or microneedle-based deliveries have been shown to yield higher immunogenicity results alongside with dose-sparing than IM^6^,^7^,^23^,^24^,^25^,^26^. Interestingly, studies have shown better immunogenicity, B and T cells responses in adjuvanted and unadjuvanted microneedle-immunised groups when compared with other cutaneous immunisation routes such as ID^25^,^27^. While the differences have not been fully understood, efficiency of antigen uptake and danger signal release from the vaccination device may contribute to the observed variation in immunogenicity. QA adjuvanted groups yielded at least 3-fold higher CD8^+^ T cells responses to ovalbumin compared to unadjuvanted groups when immunised with the Nanopatch^7^. Similarly, when antigen was co-delivered with QA using the Nanopatch, it reduced the antigen dose down to 900-fold lower than IM to induce similar antibody responses^24^. Together, the combination of adjuvants and Nanopatch skin delivery is a promising platform to deliver antigens to achieve robust immune responses.

While QS-21 is a safe and efficacious adjuvant, pain and tenderness associated with QS-21 at the site of immunisation has been observed at 50 and 100 μg 10, doses routinely used in IM vaccinations in humans. IM vaccination studies using the needle and syringe with human volunteers demonstrated mild to moderate pain^10^ and 17% with grade III symptoms (prevention of normal everyday activities)^14^ in two separate QS-21 related studies. Even though pain scoring is subject to an individual’s pain threshold, QS-21 associated pain was adequate to deter some individuals from continuing the vaccination regime^28^. The pain was reported to occur immediately upon withdrawal of the needle, minutes later and could generally last for hours to days^14^,^28^. This pain could be due to either the high dose of QS-21 (i.e. 50 or 100 μg)^10^,^14^ or the delivery method (i.e. needle and syringe IM delivery)^29^,^30^ or the combination of both.

Besides immunological advantages, Nanopatches could also potentially further increase patient acceptability of QS-21 based vaccine formulations because the optimal adjuvant dose required is much lower than that required for IM vaccinations, inducing optimal antibody response (1.5 μg versus 50 μg in mouse model). By offering a needle-free targeted delivery method to skin that is minimally invasive and requires low doses, the Nanopatch has the potential to reduce the pain of QS-21 administration. Typical microneedles have been reported to induce significantly less pain than a conventional 26-gauge needle and syringe, and decreasing the length of microneedles (from 1450 μm to 480 μm) further reduces pain^29^,^30^. The microprojections on the Nanopatch (around 110 μm long) are shorter than typical microneedles, and therefore less likely to interact with pain receptors in the skin, which are predominantly deeper in the dermis^31^. Furthermore, we demonstrated that the Nanopatch only required a low adjuvant dose (0.5 to 3.0 μg)^7^,^24^ to induce robust immune response compared to 20 to 50 μg of QS-21^32^,^33^ in mouse model. Thus, using the Nanopatch delivery system could further improve patient acceptability by diminishing pain inducing factors (i.e. reduced dose of QS-21 and targeting superficial skin layers) when used with QS-21 adjuvant compared to deep IM vaccinations.

Here, we investigated the combination of Nanopatch and the purified adjuvant QS-21 as a platform for enhancing antibody-mediated immune responses. We examined the adjuvant action of QS-21 delivered into the skin with the Nanopatch, co-administered with a test case human seasonal influenza antigen (Intanza 2013).

## Results

### The enhancement of various antigen specific IgG subtypes in Nanopatch application is QS-21 dose dependent

**Figure 1:**
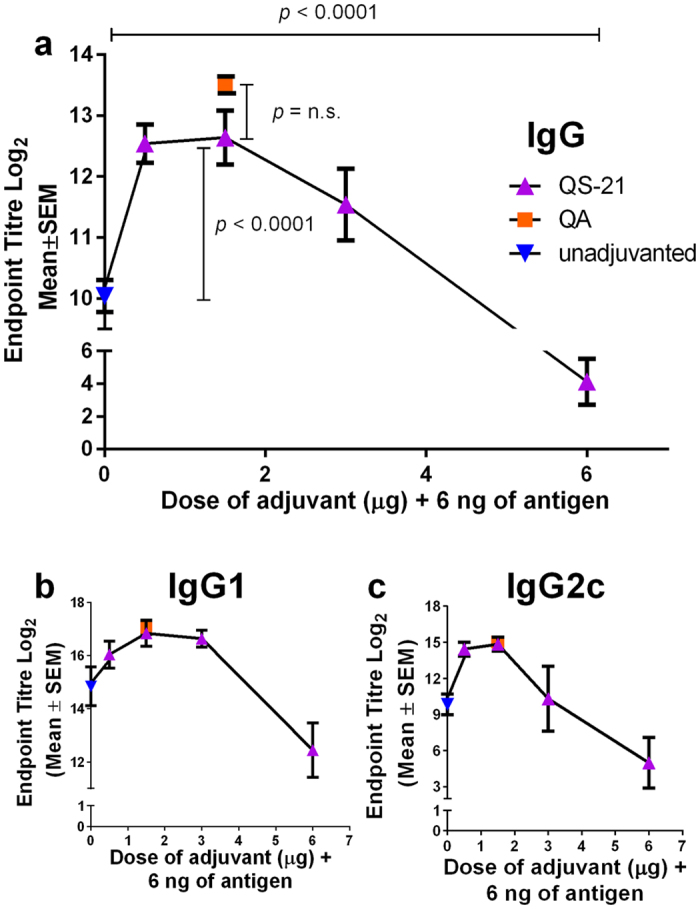
Adjuvant dose curve of antigen specific IgG, IgG1 and IgG2c responses induced by influenza antigen and co-delivery of different dose of QS-21 or 1.5 μg of QA by Nanopatch. Nanopatch groups were represented by 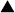 (unadjuvanted 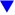, QS-21 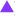) and 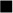 (QA 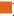) (**a**) Total antigen specific IgG (**b**) IgG1 and (**c**) IgG2c induced by co-administering 6 ng of influenza antigen with QS-21 at different doses (0, 0.5, 1.5, 3.0 and 6.0 μg) or 1.5 μg QA delivered by the Nanopatch, 21 days post immunisation. ELISA antibody data represent the Mean ± SEM, statistical significance is when *p* < 0.05, total antigen specific IgG was performed with two independent sets of n = 5 C57BL/6 mice per group, IgG1 and IgG2c are performed with one set of n = 5 mice per group. Statistical tests between three or more groups were performed with one-way ANOVA and between two groups were performed using Student’s t-test.

We have previously shown using the adjuvant QA that Nanopatch delivered vaccine induced a strong antigen specific IgG response^24^. We have used 6 ng of influenza antigen as determined previously (Supplementary Figure 1), showing detectable antigen specific IgG from a low dose of antigen induced by the Nanopatch. In Fig. 1, a range of doses of QS-21 (0, 0.5, 1.5, 3 and 6 μg) was investigated to determine the optimal dose in Nanopatch as assessed by IgG, IgG1 and IgG2c ELISAs on sera day 21 post immunisation. We also included QA as the control and compared the differences between QA and QS-21. As shown in Fig. 1, dose matched Nanopatch groups with either adjuvant, QA or QS-21, induced similar levels of antigen specific IgG, IgG1 and IgG2c (*p* *=* 0.071, n.s., *p* = 0.725, n.s., *p* *=* 0.785, n.s.). Both adjuvants, QA and QS-21, enhanced both Th1 and Th2 responses compared to unadjuvanted groups and were in agreement with studies by others^34^,^35^. A similar trend between antibody responses and QS-21 dose was observed, where an increase in antibody response correlated with an increase of administered QS-21 doses, peaking at 1.5 μg, significantly higher in antigen specific total IgG (*p* < 0.0001; Fig. 1). A further increase of QS-21 dose led to a decrease in antibody responses. Therefore, the optimal dose of QS-21 was determined to be 1.5 μg when delivered with the Nanopatch. Taken together, these results suggested a balanced antigen specific IgG, IgG1 and IgG2c response post 21 days after vaccination with QS-21 adjuvant at an optimal dose of 1.5 μg.

### IM immunisation of QS-21 enhanced various antigen specific IgG subtypes responses and resulted in a balanced Th1/Th2 response

**Figure 2:**
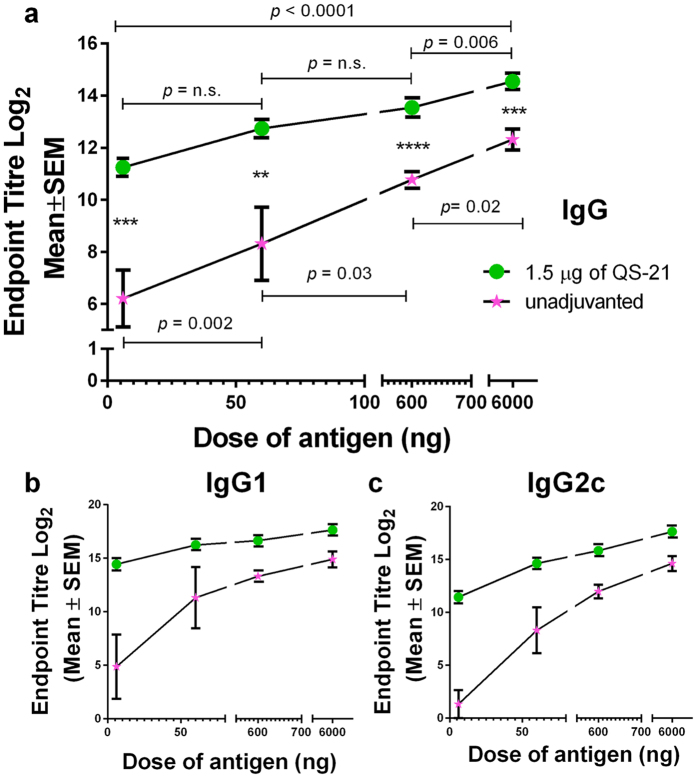
Antigen dose curves of antigen specific IgG, IgG1 and IgG2c responses induced by influenza antigen and with or without 1.5 μg of QS-21 by the needle and syringe intramuscularly (IM). IM groups were represented by 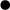 and 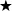 (unadjuvanted 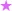 and with 1.5 μg of QS-21 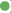). (**a**) Total antigen specific IgG (**b**) IgG1 and (**c**) IgG2c induced by co-administering of influenza antigen different doses (6, 60, 600, and 6000 ng) unadjuvanted or with 1.5 μg of QS-21 by IM, 21 days post immunisation. ELISA antibody data represent the Mean ± SEM, statistical significance is when *p* < 0.05, *p < 0.05; **p < 0.005; ***p < 0.0005; ****p < 0.001, total IgG was performed with two independent sets of n = 3 to 5 C57BL/6 mice per group, IgG1 and IgG2c were performed with one set of n = 3 to 5 per group. Statistical tests between three or more groups were performed with one-way ANOVA and between two groups were performed using Student’s t-test.

Previous studies with QA and/or QS-21 reported an enhancement in both Th1 and Th2 responses with IM delivery^9^,^15^,^32^,^34^. In Fig. 2, we measured IM induced antigen specific IgG, IgG1 and IgG2c responses after immunising mice with a range of doses of influenza antigen (6, 60, 600 and 6000 ng), with and without 1.5 μg of QS-21. Overall, QS-21 enhanced antigen specific IgG, IgG1 and IgG2c titres compared to unadjuvanted control groups at all vaccine doses tested. A sharp increase of IgG, IgG1 and IgG2c titres was observed with increasing vaccine dose from 6 to 60 ng (Fig. 2). A more gradual increase of IgG subtypes was observed beyond 60 ng. This suggested that a considerable amount of vaccine or an addition of adjuvant was essential for IM immunisation to induce a stronger IgG response. Co-administration of QS-21 in IM immunisations resulted in a more balanced Th1-Th2 response, which corresponded well with the literature^16^,^35^. In summary, QS-21 enhanced antigen specific IgG responses and IgG subtypes in response to IM vaccination.

### Nanopatch induced higher IgG compared to IM, with or without QS-21

**Figure 3:**
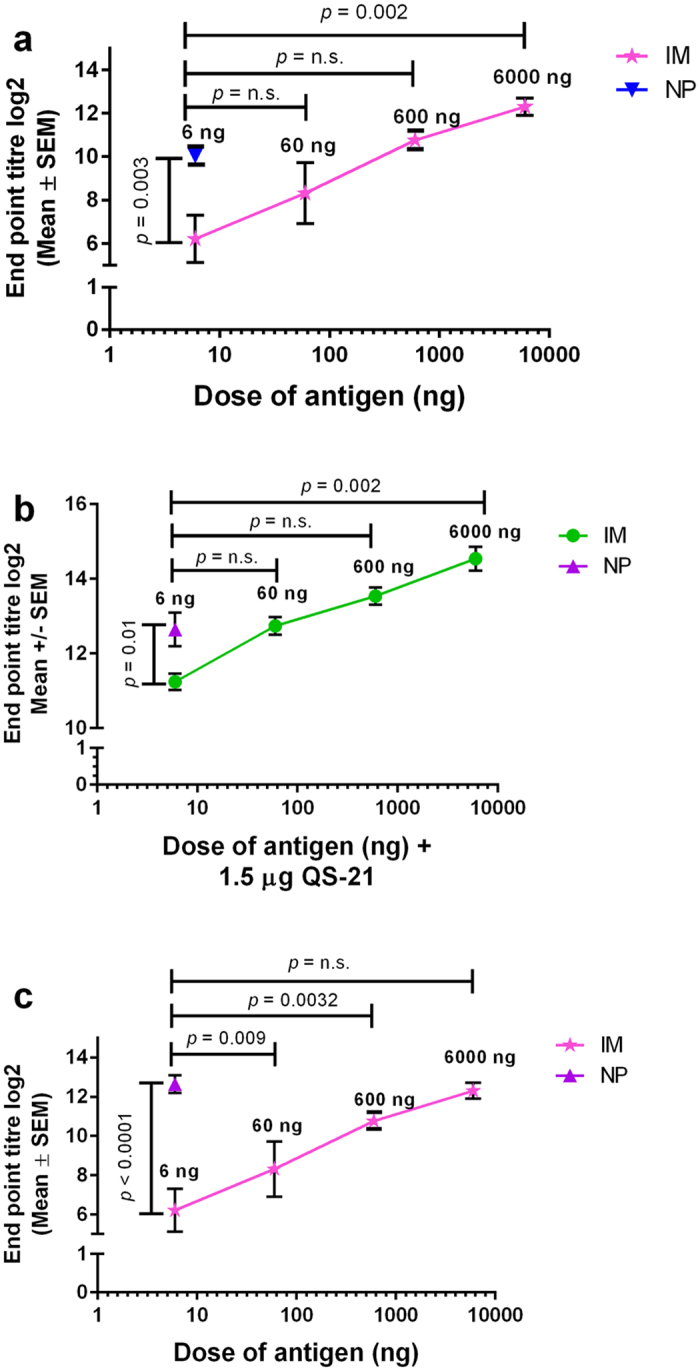
Total serum antigen specific IgG response comparing influenza antigen doses with/without 1.5 μg of QS-21 day 21 day post immunisation delivered by Nanopatch or needle and syringe intramuscular (IM) route. Nanopatch groups were represented by 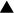 (unadjuvanted 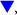, QS-21 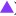), IM groups were represented by 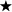 and 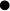 (unadjuvanted 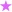 and of QS-21 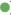). (**a**) Total serum antigen specific IgG was unadjuvanted influenza antigen, delivered by Nanopatch (at 6 ng) and IM (at 6, 60, 600 and 6000 ng). (**b**) Total serum antigen specific IgG was co-delivered with 1.5 μg of QS-21 and influenza antigen, delivered by Nanopatch (at 6 ng) or IM (at 6, 60, 600 and 6000 ng). (**c**) Total serum antigen specific IgG was co-delivered with 1.5 μg of QS-21 and influenza antigen, delivered by Nanopatch (at 6 ng) compared to unadjuvanted IM (at 6, 60, 600 and 6000 ng). ELISA antibody data represent the Mean ± SEM, statistical significance is when *p* < 0.05, total IgG was performed with two independent sets of n = 3 to 5 C57BL/6 mice per group. Statistical tests between two groups were performed using Student’s t-test.

We previously demonstrated that using the Nanopatch delivery system, an equivalent antigen specific IgG was achieved compared to IM with 100-fold dose reduction^6^. With the addition of QA to Nanopatch, the dose reduction increased to 900-fold when compared to unadjuvanted IM^24^. To investigate whether QS-21 would achieve similar effects of increasing dose reduction when compared to IM delivery as QA as observed in previous studies^6^,^24^, we immunised mice with 6 ng of antigen by Nanopatch and a range of antigen (6, 60, 600 and 6000 ng) by IM, with or without 1.5 μg of QS-21. We assessed the unadjuvanted (Fig. 3a) or QS-21 adjuvanted (Fig. 3b) antigen specific IgG with different dose of antigen delivered by either IM or the Nanopatch. A comparison between adjuvanted Nanopatch and unadjuvanted IM was also performed (Fig. 3c). Overall, similar levels of dose sparing were elicited for both unadjuvanted and QS-21 groups.

Specifically, we found that the unadjuvanted Nanopatch group required 100-fold less dose than IM to induce similar antigen specific IgG response (Fig. 3a, *p* *=* 0.062, n.s.). Upon addition of QS-21, Nanopatch delivered antigen maintained the level of dose reduction, suggesting that QS-21 enhanced antigen specific IgG production following Nanopatch and IM immunisations into the skin and muscle, respectively. Separately, 6 ng of antigen delivered by Nanopatch induced an equivalent level of antigen specific IgG to 600 ng induced by IM (Fig. 3b, *p* *=* 0.091, n.s.). Together, these results demonstrated that skin based delivery by Nanopatch was more efficient in inducing antigen specific IgG than IM, regardless the addition of QS-21. We have compared QS-21 adjuvanted Nanopatch vaccination with the standard IM unadjuvanted vaccination as a reference. The results (Fig. 3c) demonstrated equivalent antigen specific IgG levels were induced in both cases, therefore, an about 1000-fold dose sparing observed with the Nanopatch QS-21 conditions (NP 6 ng with 1.5 μg QS-21 versus IM 6000 ng; *p* *=* 0.592, n.s.).

With a significant dose-sparing of Nanopatch over IM, we next sought to investigate the quality of antigen-specific IgG (avidity of IgG binding to the antigen). The avidity assay showed that skin based delivery induced high avidity IgG while co-administration of QS-21 did not further increase IgG avidity (Supplementary Figure 2b(i,ii)). Specifically, Nanopatch groups induced IgG avidity of 59.4% ± 4.9%, while co-delivery of 1.5 g of QA enhanced the avidity to 62.2% ± 4.7% whereas co-administration of 1.5 μg of QS-21 resulted in an avidity of 58.6% ± 6.9%, no statistical significance were found between all Nanopatch groups (Supplementary Figure 2b (i,ii)). This is indicative that the Nanopatch had already induced high avidity IgG and an addition of adjuvant did not help in enhancing IgG avidity. However, QS-21 improved the avidity of antigen specific IgG significantly in adjuvanted IM immunisations beyond 60 ng of antigen – 600 ng increased from 39.4% ± 5.1% to 74.5% ± 7.5% (*p* *=* 0.0161) and 6000 ng increased from 68.8 ± 3.2% to 91.0% ± 3.4% (*p* *=* 0.0033) (Supplementary Figure 2c (iii,iv)). Delivering 6 ng of antigen in unadjuvanted Nanopatch groups resulted in significantly higher avidity than 600 ng unadjuvanted IM groups, with 59.4% ± 4.9% and 39.4% ± 5.1%, respectively (*p* *=* 0.0383) (Supplementary Figure 2a). Taken together, the Nanopatch induced high magnitude and avidity IgG regardless of an addition of QS-21, while IM required either a higher antigen dose or an addition of adjuvant to induce equivalent response, resulting in a 100-fold dose reduction when comparing Nanopatch to IM. With the addition of QS-21, Nanopatch induced an equivalent antigen specific IgG with unadjuvanted IM, indicative of about 1000-fold dose sparing effect.

### Nanopatch immunisation requires only low antigen and QS-21 doses compared to IM injection

Compared to IM delivery, Nanopatch delivery efficiently induced higher antigen specific IgG, when QS-21 is co-administered with the antigen. Here we investigate whether the dose of QS-21 co-delivered with IM could induce comparable antigen specific IgG as the Nanopatch, and whether increase in antigen doses of Nanopatch will further increase the antigen specific IgG levels. Two doses of QS-21 (either 10 or 50 μg) were co-delivered with ranged doses of influenza antigen (6, 60, 120 and 600 ng) by IM. Separately, an increasing dose of influenza antigen (6, 60 and 120 ng) was co-delivered with 1.5 μg of QS-21 (optimal QS-21 dose for Nanopatch from Fig. 1) for Nanopatch vaccination.

**Figure 4:**
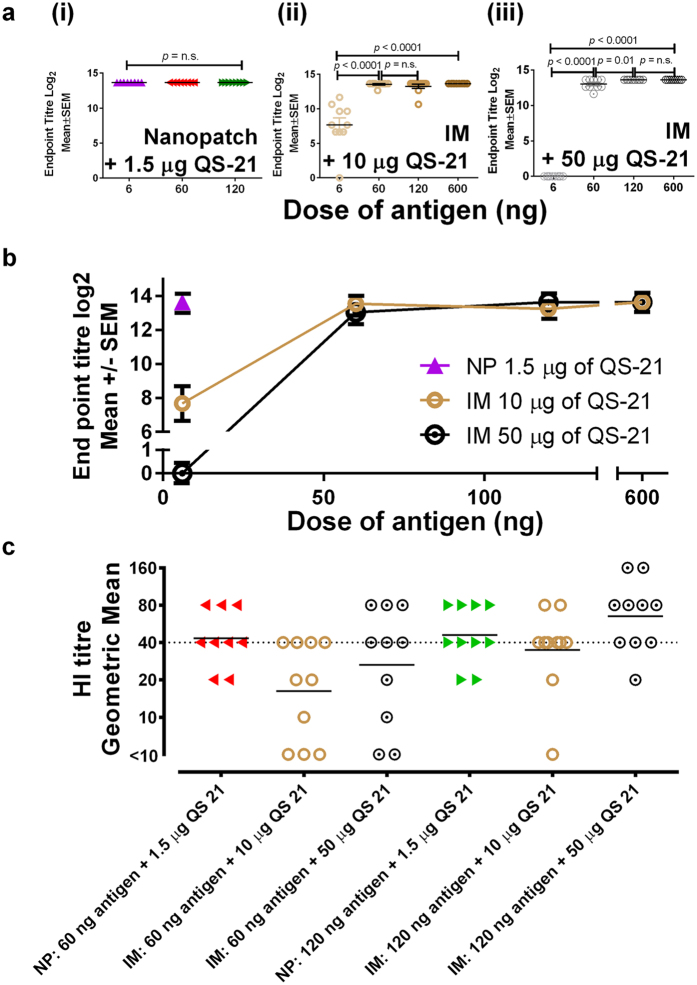
Total serum antigen specific IgG response comparing QS-21 dose 21 days post immunisation at 1.5 μg delivered by Nanopatch, or 10 μg or 50 μg delivered by the needle and syringe intramuscular (IM) route. Nanopatch groups were represented by 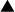 (6 ng 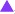, 60 ng 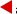 and 120 ng 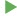 of influenza antigen) ; IM groups were represented by 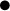 (10 μg 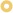 or 50 μg of QS-21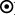) with different dose of influenza antigen (6 ng, 60 ng, 120 ng and 600 ng). (**a**) Antigen specific endpoint titres of (i) Nanopatch induced response by 6 ng, 60 ng or 120 ng of influenza antigen without QS-21, (ii) IM induced response by 10 μg of QS-21 with different dose of influenza antigen (6 ng, 60 ng, 120 ng and 600 ng, (iii) IM induced response by 50 μg of QS-21 with different dose of influenza antigen (6 ng, 60 ng, 120 ng and 600 ng; (**b**) Nanopatch and IM induce antigen specific IgG from different dose of QS-21 (1.5 μg, 10 μg and 50 μg) and (**c**) Haemagglutination Inhibition (HI) titres of one of the influenza strain A/Victoria/361/2011 (H3N2) from Nanopatch and IM at 60 ng and 120 ng of antigen with 1.5 μg, 10 μg and 50 μg of QS-21. ELISA antibody data represent the Mean ± SEM, statistical significance is when *p* < 0.05, total IgG was performed with 2 sets of n = 4 to 5 per group. HI titres data are represented by geometric mean with 2 sets of n = 4 to 5 per group. Statistical tests between 3 or more groups were performed with one-way ANOVA and between 2 groups were performed using Student’s t-test.

We found that the Nanopatch group required 30-fold less QS-21 dose than IM to induce similar antigen specific IgG response (Fig. 4b, *p* < 0.0001) at low matched antigen dose (6 ng). Specifically, no difference was observed with increased antigen dose in Nanopatch vaccination (Fig. 4a(i) and Supplementary Figure 3a), which is suggestive of the efficient uptake of antigen at 6 ng delivered by the Nanopatch to induce high antigen specific IgG. An equivalent antigen specific IgG response to Nanopatch (6 ng antigen dose with 1.5 μg of QS-21) by IM delivery required at least a dose of 60 ng of antigen and 10 μg of QS-21, demonstrating the need for higher doses of both antigen and QS-21 for IM delivery. It was observed that, at 6 ng of antigen dose, 50 μg of QS-21 induced lower total antigen specific IgG as compared to 10 μg of QS-21 for IM vaccination (Fig. 4b, *p* < 0.0001), which is indicative that excessive QS-21 is detrimental to the induction of antigen specific IgG, which is in agreement with the literature^28^. At higher antigen dose, 10 and 50 μg of QS-21 induced similar antigen specific IgG (Fig. 4a(ii,iii), Supplementary Figure 3b,c).

Haemagglutination inhibition (HI) assay was performed as a surrogate of protection at 63 days post immunisation (Fig. 4c). Here, we tested one of the influenza antigens, A/Victoria/361/2011 (H3N2)-like strain with HI assay (Fig. 4c). A HI titre of 40 and above for naïve humans will be considered as protective^36^, hence, we assumed similar HI titres for protection in mice. At matched antigen dose of 60 ng, Nanopatch demonstrated HI titre of more than 40 at 1.5 μg of QS-21, but QS-21 adjuvanted IM doses (10 or 50 μg) achieved HI titres of less than 40. However at higher antigen dose of 120 ng, HI titre beyond 40 is only observed with IM delivered QS-21 at 50 μg. Nanopatch delivered QS-21 at 1.5 μg was consistent in inducing HI titres of more than 40 at all antigen doses tested. This demonstrated the 30-fold adjuvant dose sparing effects of Nanopatch as compared to IM.

Taken together, IM route required higher antigen and adjuvant dose when inducing equivalent antigen specific IgG as opposed to Nanopatch which required a low antigen and adjuvant dose, is indicative that the Nanopatch is an efficient delivery method for immunisation requiring lesser antigen and adjuvant doses for protective effects.

### QS-21 adjuvant increases cellular death in skin, thereby enhancing the systemic immune responses

**Figure 5:**
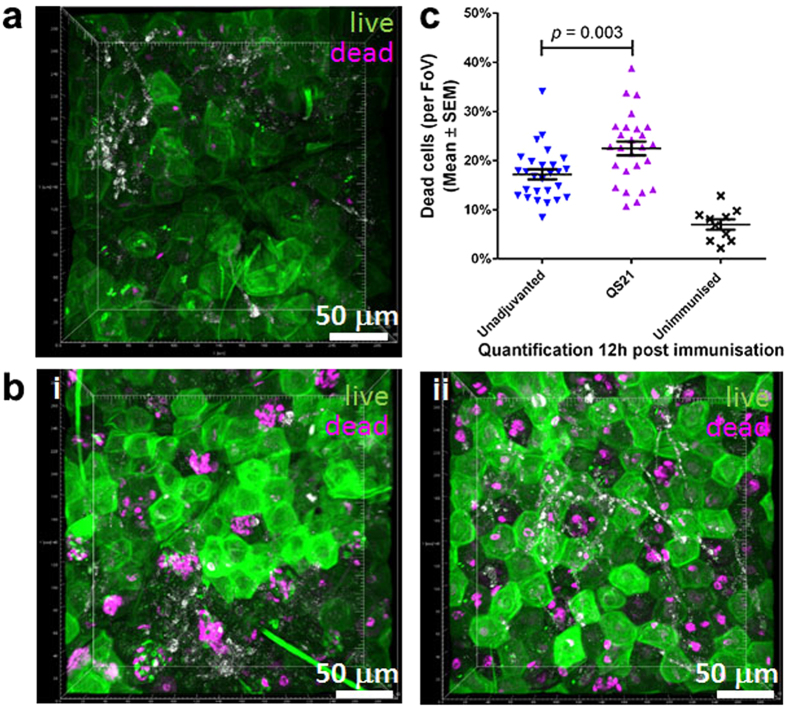
Increased cell death post Nanopatch application co-delivered with QS-21. Nanopatch groups were represented by 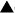
(unadjuvanted 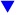, QS-21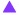) and × unimmunised group. Cellular viability at 12 h post Nanopatch application ± QS-21 was imaged by Multiphoton Microscopy. Ear skin samples from (**a**) untreated and, (**b**) immunised with 6 ng of influenza vaccine (i) unadjuvanted or (ii) adjuvanted with 1.5 μg QS-21. (**c**) Quantification of cellular viability resulted in significantly higher amount of cell death with QS-21 adjuvant (17.2% ± 5.3–22.5% ± 7.1%). Magenta represents dead cells and green represent live cells. Images are representative of n = 5 C57BL/6 mice (Nanopatch groups) or n = 3 (naïve) with up to 5 measurements per mouse). Statistical tests between 2 groups were performed using Student’s t-test.

With the Nanopatch known to cause localised cell death that has been correlated to enhanced immunogenicity^27^, and QS-21 shown to induce cell lysis^8^,^37^, we next sought to investigate whether QS-21 would increase cell death in skin in the vicinity of the microprojections of the Nanopatch. We assessed the skin vaccination sites 12 hours post-delivery of unadjuvanted and QS-21 adjuvanted influenza vaccine groups using a viability stain. *In situ* imaging of cellular viability^27^ showed low levels (6.9% ± 3.3%) of dead cells in untreated skin (Fig. 5a). In contrast, vaccinated unadjuvanted groups showed a clear pattern of dead cells that corresponded well with the Nanopatch microprojections penetrating the skin (Fig. 5b(i)), resulting in 17.2% ± 5.3% of cell death. With QS-21 adjuvanted group, a statistically significant increase in cell death was observed (22.5% ± 7.1%) when compared to unadjuvanted groups (Fig. 5b(ii)), which is summarised in Fig. 5c (*p *= 0.003). Together, these findings suggest that QS-21 evokes further cell membrane damage, resulting in more localised cell death in murine ear skin post Nanopatch applications, which could lead to higher immune response.

## Discussion

We have shown, for the first time, the use of a single low QS-21 dose at 1.5 μg delivered to skin by Nanopatches with a licensed influenza subunit antigen induces robust antigen specific IgG responses in a mouse model (measured 21 days post immunisation). In contrast, delivery of QS-21 by IM to mice required at least 10 to 50 μg of QS-21, with a combination of other adjuvants, to achieve robust immune responses^32^,^33^,^38^. A scaled up dose of 50 to 100 μg of QS-21 and 50 μg MPL (AS01/AS02 adjuvant mixtures) were required in human clinical trials to achieve robust immune responses with IM delivery^13^,^14^,^16^. We have shown the usage of low adjuvant dose to achieve strong humoral antigen specific IgG response (observed in Fernando *et al*.^6^ and Fig. 1), as well as for cellular CD8^+^ T cell response with QA in our previous study^7^, when delivered into mice skin with the Nanopatch. Similarly, antigen doses delivered with the Nanopatch were greatly reduced to attain similar IgG responses as IM groups (Fig. 3a,b; 6 ng with the Nanopatch compared to 600 ng with IM), and with the addition of an adjuvant, QS-21, to Nanopatch, the dose sparing escalates up to 1000-fold dose sparing (Fig. 3c), which was in agreement with our previous studies^6^,^24^.

It has been shown that the magnitude of IgG titres is increased by co-administration of adjuvants^16^,^39^. However, work by others has been largely focussed on standard IM injection – we explored the mechanism of QS-21 when delivered with the Nanopatch into the skin or IM. Antigen specific IgG, IgG1 and IgG2c titres increased when QS-21 was co-administered by Nanopatch (Fig. 1) and IM vaccination (Fig. 2). By increasing the dose of the adjuvant or antigen, an increase immune response, up to a certain threshold, is usually observed^40^. A similar trend was seen here in both adjuvanted and unadjuvanted IM groups with a gradual increment in antigen specific IgG, IgG1 and IgG2c titres (Fig. 2). Distinctively, Nanopatch delivery yielded a biphasic trend, with a gradual increment and peak at QS-21 dose of 1.5 μg, while higher adjuvant dose led to a decrease in antigen specific IgG, IgG1 and IgG2c titres (Fig. 1). This trend was observed previously with other adjuvants (i.e. QA and CpG ODN) when doses were varied to investigate CD8^+^ T cell responses induced by the Nanopatch^7^. A study using QS-21 and rsgp120 (HIV subunit protein antigen) to vaccinate human volunteers via IM also observed a biphasic trend of neutralisation antibodies^28^. The highest neutralisation antibody response was achieved at 50 μg of QS-21, while a range of 0, 50, 100 μg of QS-21 co-administered with a 100 μg of antigen resulted a biphasic trend^28^. We speculated that the occurrence of this biphasic trend in Nanopatch groups may be related to the delivery of vaccine with the Nanopatch to the immediate vicinity of APCs (imaged in Fernando *et al*.^6^ and further investigated in Raphael *et al*.^20^). Specifically, the increased dose of QS-21 might have an unfavourable cytolytic effect that could be too toxic for skin delivery resulting in the reduced IgG titres (Fig. 1 and Evans *et al*.^28^). To the best of our knowledge, other studies have not investigated skin-based delivery and thus corroborate further skin-based immunisation studies using QS-21.

Some adjuvants, such as GM-CSF^41^ and MF59^42^, are known to induce higher avidity IgG when immunised by IM. This corresponded well with our study, when QS-21 was co-administered to all doses of influenza vaccine with IM. However, in Nanopatch immunisations, QS-21 did not increase the avidity of IgG (Supplementary Figure 2b(i,ii)). This may be due to the fact that the Nanopatch application already induced a strong physical immune enhancer effect as described previously^27^, and that a combination of physical and chemical adjuvants may not always work synergistically or have already peaked the positive adjuvantation threshold. This was based on the observations that the addition of QS-21 did not alter the avidity of IgG in Nanopatch vaccination, but significantly enhanced in IM vaccination with antigen doses above 60 ng (Supplementary Figure 2c(iii,iv)). Even without QS-21, 6 ng by Nanopatch induced higher avidity IgG than IM at 6 to 600 ng of antigen and similar avidity IgG was induced when compared to IM at 6000 ng of antigen (Supplementary Figure 2a). A possible explanation may lie in the different resident immune cells and proportion/kinetics of infiltrating immune cells in the different tissue types of skin and muscle that could contribute to downstream activation of chemokines/cytokines leading to enhanced immune response^43^. However, the presence of QS-21 or QA did not affect the avidity of IgG induced by Nanopatch (Supplementary Figure 2b(i,ii)), which may be explained by the low dose of antigen/QS-21, action of the antigen/QS-21 or the single vaccination regime used in this study. Others have shown direct relationship between dose of antigen/adjuvants and avidity of IgG, by immunising a higher dose of antigen/adjuvant or multiple boosters regime resulted in an increased of IgG avidity^39^,^41^,^44^,^45^.

Th1 and Th2 type immunity can be differentiated in two ways, antigen-specfic IgG subtypes analysis or measuring the production of cytokines such as, IL-4 and IFNγ^46^. We demostrated an increase of both IgG1 and IgG2c upon the addition of QS-21 in both Nanopatch (Fig. 1b,c) and IM (Fig. 2b,c) groups. However, the lack of difference in IL-4 and IFNγ production (Supplementary Figure 4) could be attributed to the lack of strong CD4^+^ or CD8^+^ T cells epitopes in influenza HA protein in C57BL/6 mice that is restricted by H-2^b47^. Similar limitation was observed by Zhong *et al*.^47^, identifying that the influenza virus (PR8) major CD8^+^ T cells epitopes were located in the nucleoprotein and polymerase acidic protein, and not in the HA protein. However, we used purified HA proteins from A/California, A/Victoria and B/Wisconsin as the antigens in this study. Because the CD8^+^ T cells epitopes in these HA proteins may also be very weak^47^, an alternative approach developed by Ingulli *et al*.^48^, using ovalbumin attached to neuraminidase stalk protein expressing influenza virus to infect their mouse models and the strong CD8^+^ T cells epitope from ovalbumin (SIINFEKL) was used for re-stimulation to detect as a surrogate for the influenza specific CD8^+^ T cell responses. Repeating our study using Inguilli *et al*.’s approach may have be an interesting option to verify our results. However, we did not have access to the engineered ovalbumin construct to use in our study. Furthermore, others have shown that skin/muscle vaccination with QS-21 adjuvant induced strong antibody^14^,^25^,^32^ and cellular response^12^,^16^. We have previously demostrated that the Nanopatch induced strong antibody and cellular response with other antigen^6^,^7^,^24^,^27^. Thus, in this study, even though a correlation could not be drawn from the IgG subtypes and cytokine production, the observed discrepancy would likely be due to the technical limitation of insufficient HA stimulation instead of the vaccine formulation or the adminstration route.

Antigen dose sparing has been demonstrated with various vaccines multiple times with the Nanopatch in previous^6^,^7^,^21^,^27^,^49^ and current studies. Dose sparing of antigen is cost effective, crucial for the safety of the vaccine recipient and demonstrates the efficiency of the vaccine. It is important to receive sufficient vaccine for the induction of immune response and not to exceed the maximal safe dose. Similar attributes applies for adjuvants as well, due to its composition, adjuvants can cause adverse effects^50^,^51^. Adverse effects such as pyrexia, muscle weakness, arthralgia, myalgia and erythema were found following vaccines containing adjuvants^50^. However, the addition of adjuvants assists in the enhancement of immune response. There is a need to draw a clear distinction between acceptable range of side effects and optimal immune response^52^. Previously, Nanopatch with QA demonstrated an equivalent antibody response to influenza vaccine when compared to IM resulted in up to 900-fold antigen dose sparing effects. In current study, we investigated the ability for adjuvant dose sparing, which could potentially be a solution to counter the problem of adverse effects due to high dose adjuvant regime.

A fixed dose of QS-21 resulted in a constant antigen specific IgG response (Fig. 4a(i) and Supplementary Figure 3a), as opposed to QA with an increasing IgG levels, when co-delivered with an increasing dose of antigen^24^ using the Nanopatch. This could be potentially due to the differences in the manufacturing of the antigen strains, dose of antigen (highest dose used in this study for Nanopatch was 120 ng compared to previous QA study with highest antigen dose at 270 ng^24^) or the purity of the QS-21 adjuvant^9^. Wang *et al*.^53^ also noted that slight difference in composition (4 antigen strains versus 3 antigen strains) of the influenza vaccine could have different rate of seroconversion of each antigen strain, supporting our findings. Significant increase of antigen specific IgG in IM is observed in either a low antigen dose range (6–120 ng) or high QS-21 dose (50 μg) (Fig. 4a(ii,iii)). This is indicative of the need for high antigen dose, or a high QS-21 dose, or a combination of both, to achieve a significant increase of IgG response, in the case of IM delivery.

Nanopatch has shown an at least 30-fold QS-21 dose sparing effect as opposed to IM vaccination at 6 ng antigen dose (Fig. 4b). Specifically, Nanopatch at 1.5 μg of QS-21 induced higher antigen specific IgG than IM at 10 μg (*p* = 0.0002) or 50 μg (*p* < 0.0001) of QS-21 when co-administered with 6 ng of antigen. By increasing the antigen dose in IM to 60 ng, IM induced equivalent antigen specific IgG to Nanopatch at 1.5 μg of QS-21 and 6 ng of antigen (Fig. 4b; IM at 10 μg of QS-21, *p* = 0.35, n.s.; IM at 50 μg of QS-21 *p* = 0.17, n.s.). HI titre of more than 40 was observed in groups with QS-21 dose of 50 μg with 120 ng of antigen for IM delivery and 1.5 μg of QS-21 in Nanopatch at 60 or 120 ng of antigen (Fig. 4c). This further demonstrated that Nanopatch is more efficient in inducing antigen specific IgG than IM and IM required high antigen and adjuvant dose to induce HI titres beyond 40. The Nanopatch could potentially alleviate the side effects of QS-21 by using a lower antigen and adjuvant dose, without reducing the response of antigen specific IgG.

Dead cells have been reported to elicit sterile/semi-sterile inflammation via release of endogenous adjuvants including danger signals to attract inflammatory cells and increase the immune responses^54^,^55^. Physical adjuvantation resulting in enhanced immunogenicity has been reported by Depelsenaire *et al*. after Nanopatch application^27^. Similarly, others have found localised adjuvantation with laser treatment prior to typical microneedle application and needle-based intradermal injections^25^. It was postulated that the release of danger signals (such as DNA, ATP, uric acid, etc.) were correlated with the observed increased immunogenicity^25^,^27^,^54^. Indeed, Vono *et al*. found that ATP release from muscle was essential for adjuvanticity of MF59 when immunised via IM^56^. We also observed cell death in unadjuvanted Nanopatch groups as reported previously^27^. While we did not assess for danger signals, it is likely that endogenous danger signals stimulated the immune system post Nanopatch application. This will be further investigated in subsequent studies. QS-21’s haemolytic ability further contributed to the higher cell death levels measured in skin when compared with unadjuvanted Nanopatch group (Fig. 5c). However, previous study demonstrated that elevated levels of cell death caused by multiple Nanopatch applications reduced the antigen specific IgG titres^27^. Similar findings with QS-21 were also observed in this study, where an increasing amount of QS-21 reduced the antigen specific IgG; although it must be noted that no viability experiments of varying QS-21 concentrations were performed. These findings suggested that too much of either physical^27^ or chemical adjuvant (Fig. 1) can potentially be detrimental to elicit antigen specific IgG because of the extensive cellular damage. While the current mechanism of this immune enhancement is not fully understood, we speculated that the extended region of dead cells might be releasing danger signals attracting infiltration of inflammatory cells. The release of danger signals may potentially work synergistically with cytokine/chemokine production, recruiting neutrophils to the site of immunisation as early as 16 hours post vaccination^57^. Further studies should be performed to understand the importance of immunological cell types in the induction of antigen specific IgG post Nanopatch immunisation.

Overall, this study demonstrated a targeted vaccine delivery into skin has the potential to induce high titres and avidity IgG, with improved acceptability as a result of dose sparing effect of both the antigen and adjuvant.

## Materials and Methods

### Animals

All methods performed in this study were carried out in accordance with National Health & Medical Research Council (NHMRC) guidelines and approved by The University of Queensland Animal Ethics Committee. All animal care and experiments were conducted in accordance with NHMRC (Australia) guidelines and with the approval of The University of Queensland animal ethics committee under AIBN/556/12/ARC/NHMRC/SMART. Two independent experiments, unless specified, with groups of n = 3 to 5 female C57BL/6 J mice of 6 to 8 weeks were used per condition, and they were maintained under specific pathogen-free condition in the animal facility of Australian Institute of Bioengineering and Nanotechnology, The University of Queensland. All experiments were repeated at least two times to ensure that the results were reproducible.

### Vaccine formulation and Immunisation

Intanza 2013 (Sanofi Pasteur) is a trivalent influenza subunit protein vaccine containing 90 μg/ml of each influenza strains, A/California/7/2009 (H1N1)pdm09-like strain, A/Victoria/361/2011 (H3N2)-like strain and B/Wisconsin/1/2010-like strain.

The Nanopatches were manufactured as described elsewhere^58^, measuring 4 by 4 mm with a density of 21,000 microprojections per cm^2^, and microprojections were 110 μg long. The Nanopatches were cleaned with 70% ethanol, rinsed with MilliQ water and air dried. The Nanopatch vaccine formulation consisted of 1% methylcellulose (Methocel 60 HG; 64655; Fluka/Sigma-Aldrich), 5 ng of Intanza 2013 and Dulbecco’s Phophate-Buffered Saline (dPBS, 14190-144; Gibco/Life Technologies) as the diluent. Vaccine formulation was dry coated on to the Nanopatch by a 70° and 20° angle nitrogen gas jet as described previously^59^. One Nanopatch per mouse was used to vaccinate mice with an spring loaded applicator at a constant velocity of 1.8 m/s to penetrate microprojections on the Nanopatch into the ear skin of mice^19^ and left for 2 minutes to allow the reconstitution and diffusion of the dry coated vaccine^20^. The amount of vaccine delivered into the skin was quantified using a radioactive tracer^24^.

IM injections were prepared by diluting Intanza 2013 in dPBS to the appropriate dose (6, 60, 600 and 6000 ng). IM injections were administered to one side of the thigh caudal muscle (25 μl) per mouse.

### Sample collection

Blood was collected by retro-orbital bleeds on day 21 and terminal bleeds on day 63. Blood was kept at room temperature for 2 hours for clotting before centrifugation at 10,000 g for 5 minutes to separate sera from whole blood. Sera were kept at −20 °C until further analysis.

### Enzyme Linked Immunosorbent assay (ELISA)

Antigen specific antibody (day 21 sera) was measured by ELISA as previously described^4^,^5^,^6^. Briefly, 3 μg/ml of antigen (Intanza 2013) was diluted and used to coat the ELISA plates (Nunc Maxisorp, ThermoFisher) overnight at 4 °C. The plates were blocked with 0.4% BSA in PBS and used to determine the antigen specific antibody titres. Sera obtained were serially diluted and incubated for 2 hours in room temperature. Washing of plates were done 5 times using 0.02% PBST and secondary antibody, anti-mouse IgG HRP (G-21040, Invitrogen/ThermoFisher) at 1 μg/ml were added and incubated for 1.5 hours. Colour development was performed using ABTS (2,29-azino-bis3-ethylbenzthiazoline-6-sulfonic acid; A-1888, Sigma-Aldrich) as the substrate and measured at absorbance of 405 and 490 nm. Endpoint titres were calculated as described elsewhere^27^. IgG1 and IgG2c isotypes responses were assessed using the same ELISA method (IgG1, ab97240; 1:4000, and IgG2c ab97255; 1:2000; Abcam).

### Haemagglutination Inhibition (HI) Assay

Day 63 sera were treated with receptor destroying enzyme (RDE II, DenkaSeiken Co. Ltd.) prior to HI analysis to remove nonspecific inhibitors of agglutination. Serum samples were treated with RDE at 1:4 ratios and held at 37 °C overnight. A further 6 parts of PBSA (0.1 mg/ml BSA in PBS) was then added, to give a final dilution of serum of 1 in 10 and held at 56 °C for 2 hours to inactivate RDE enzyme activity. HI assay was performed on one of the three influenza antigens, A/Victoria/361/2011 (H3N2)-like strain. This assay is performed based on established methods from Victorian Infectious Diseases Reference Laboratory (VIDRL)^60^ adapted to micro-titre format using chicken red blood cells. According to US Food and Drug Administration (FDA), dilutions equal or above 1:40, an individual is considered protected^36^.

### Cell viability staining, multi-photon microscopy (MPM)

Cellular viability was assessed as previously described^27^. Briefly, ear skin was split, cartilage was carefully removed and stained using a mixture of acridine orange (0.03 mg/ml) and ethidium bromide (0.1 mg/ml), labelling live (green) and dead (magenta) cells, respectively. Positive controls were pre-treated with ice-cold methanol before staining. Imaging was performed on a LSM510 (Zeiss) using a 40x oil objective (Zeiss) and image acquisition at 1024 × 1024 pixels, with z-stacks up to 120 μm in depth at 1 μm intervals. Pseudo colours were applied while no imaging enhancing methods were used. Cell counts were performed with Imaris software x64 6.3.1 (Bitplane AG, Zurich, Switzerland) using the spot function at 4.1 μm based on quality as parameter post background subtraction.

### Statistical analyses

Statistical analyses were performed using GraphPad Prism version 6.05 for Windows (GraphPad Software, La Jolla California USA, www.graphpad.com). One way ANOVA test and two-tailed unpaired Student’s t-test were performed as appropriate with multiple comparisons or single comparison. All data represented were expressed as the Mean ± Standard error of mean (SEM) unless otherwise stated. A difference was considered statistically significant when *p* < 0.05.

## Additional Information

**How to cite this article**: Ng, H.-I. *et al*. Potent response of QS-21 as a vaccine adjuvant in the skin when delivered with the Nanopatch, resulted in adjuvant dose sparing. *Sci. Rep.* **6**, 29368; doi: 10.1038/srep29368 (2016).

## Supplementary Material

Supplementary Information
