# Supplementary material for: Potent response of QS-21 as a vaccine adjuvant in the skin when delivered with the Nanopatch, resulted in adjuvant dose sparing
Source: Sci Rep. 2016 Jul 11;6:29368. doi: 10.1038/srep29368 (PMC4941647; doi:10.1038/srep29368)
Supplement: Supplementary Information [file srep29368-s1.pdf]

Supplementary Information

Title: Potent response of QS-21 as a vaccine adjuvant in the skin when delivered with the Nanopatch, resulted in adjuvant dose sparing

Authors: Hwee-Ing Ng, Germain J.P. Fernando, Alexandra C.I. Depelsenaire, Mark A.F. Kendall

Supplementary Methods

Antibody Avidity

Avidity of IgG was determined by modifying the ELISA protocol as described, to include a 15 minutes treatment step with 6M urea after sera incubation with vaccine coated ELISA plates [42]. IgG avidity was determined for each individual post vaccination serum, absorbance from both treated and untreated groups with 6M urea was determined. Percentage of urea resistant antibodies was calculated by dividing the optical density (OD) of the urea-washed samples by the OD of the unwashed samples, and percentage below 50% was considered low avidity IgG[45].

IL-4 & IFNγ Enzyme Linked Immunospot (ELISPOT)

Interleukin-4 and interferon-gamma enzyme-linked immunospot (IL-4 and IFNγ ELISPOT) assays were performed to quantify antigen-specific cells producing IL-4 or IFNγ. This assay is well established and is based on previous study with slight modification[7]. Briefly, 7 days after vaccination, mice were euthanised by cervical dislocation and splenocytes were prepared. ELISPOT plates (Millipore MultiScreen-HA Cat#MAHAS4510) were coated with IL-4 or IFNγ capture antibody (MabTech Cat# 3311-3-250 or 3321–3; final concentration, 8 µg/ml). Influenza antigen was treated with 2.5% trypsin at 37 °C for 3 hours to release peptides. Splenocytes and treated influenza antigen (2 µg/ml) were mixed to stimulate cells in vitro at 37 °C in 5% CO_2 for 40 to 44 hours. An anti-IL-4 or anti-IFNγ biotinylated detection antibody (MabTech Cat# 3311-3-250 or 3321–6-250; final concentration, 1

μg/ml) was added and incubated for 2 hours at room temperature. Avidin horseradish peroxidase (HRP; Sigma Cat# A-3151) was then added, and the plates were incubated for 1 hour at room temperature. The substrate DAB (SigmaCat#D0426)was prepared according to the manufacturer's instructions and added to the wells until spots appeared (5 to 8 minutes).The results reported were averaged between 3 technical replicates and with the background (no peptide) counts subtracted.

Supplementary Figures

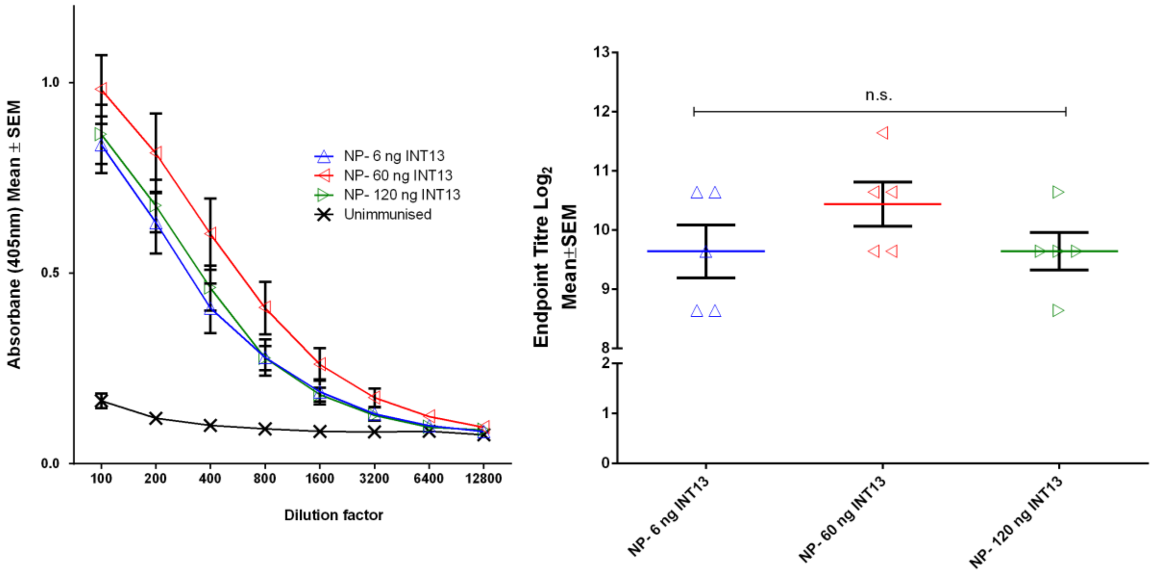

Supplement Figure 1: Antigen specific IgG day 21 post Nanopatch immunisation of C57BL/6 mice. Total serum antigen specific IgG titres from three different doses of influenza antigen represented by ▲ (6 ng △, 60 ng ◁ and 120 ng ▷) and × unimmunised mice was used as control. ELISA antibody data represent the Mean ± SEM, statistical significance is when $p<0.05$, n=5 C57BL/6 mice per group. Statistical for endpoint titres were performed with one-way ANOVA.

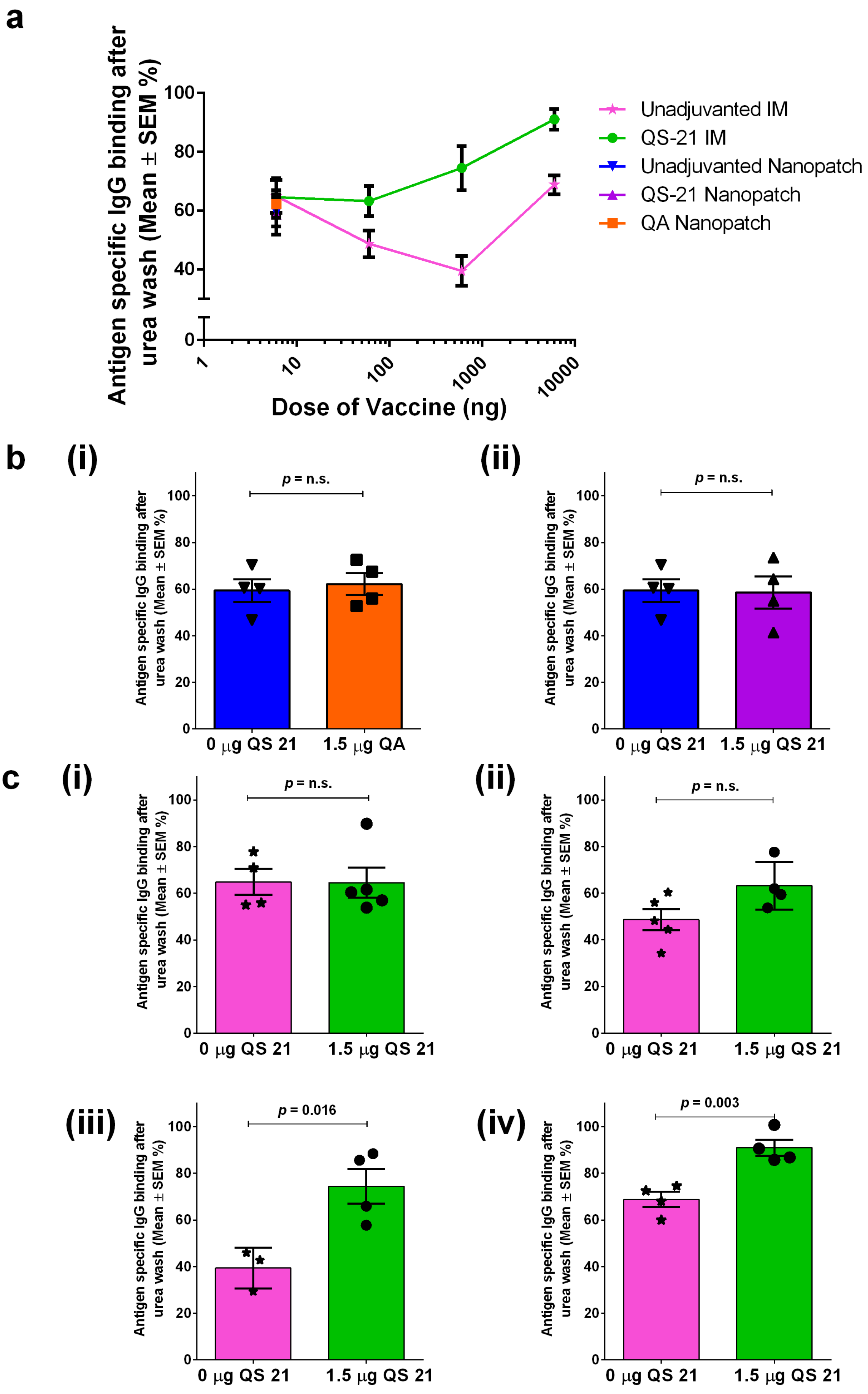
a
Antigen specific IgG binding after urea wash (Mean ± SEM %)
Dose of Vaccine (ng)
Unadjuvanted IM
QS-21 IM
Unadjuvanted Nanopatch
QS-21 Nanopatch
QA Nanopatch
b (i)
p = n.s.
0 µg QS 21
1.5 µg QA
(ii)
p = n.s.
0 µg QS 21
1.5 µg QS 21
c (i)
p = n.s.
0 µg QS 21
1.5 µg QS 21
(ii)
p = n.s.
0 µg QS 21
1.5 µg QS 21
(iii)
p = 0.016
0 µg QS 21
1.5 µg QS 21
(iv)
p = 0.003
0 µg QS 21
1.5 µg QS 21

Supplementary Figure 2: Antigen specific IgG induced by co-administering 6 ng of influenza vaccine with 1.5 µg of QA or 1.5 µg of QS-21 by the Nanopatch or co-administering 1.5 µg of QS-21 with 6, 60, 600 or 6000 ng of influenza antigen by needle and syringe intramuscular (IM) route. Nanopatch groups were represented by ▲and ■(unadjuvanted ▼, QS-21▲ and QA ■), IM groups were represented by ★ and ● (unadjuvanted ★ and of QS-21●). Avidity was assessed by studying the binding strength of the antigen specific IgG to the antigen by partially disrupting antigen-antibody interaction using a chaotropic agent, urea, by ELISA. An optimal concentration of urea will remove the low avidity binding IgG and leave the high avidity binding IgG intact. Unadjuvanted Nanopatch induced high avidity IgG (59.4%) and the addition of adjuvant did not change significantly avidity of IgG (Unadjuvanted, 59.5%; QA, 62.2%; *p* = 0.6931; QS-21, 58.6%; *p* = 0.9276). Unadjuvanted IM induced a decreasing trend of avidity IgG and then increased with increasing dose of vaccine (from 64.9% to 39.4% and then increased to 68.8%). Addition of QS-21 significantly increased the avidity of IgG, at 600 ng (39.4% to 74.5%; *p* = 0.0161) and 6000 ng (68.8% to 91.0%; *p* = 0.0033) of influenza antigen. ELISA IgG binding % data represent the Mean ± SEM, statistical significance is when p<0.05, n= 3 to5 per group. Statistical test for endpoint titres were performed with Student t-test.

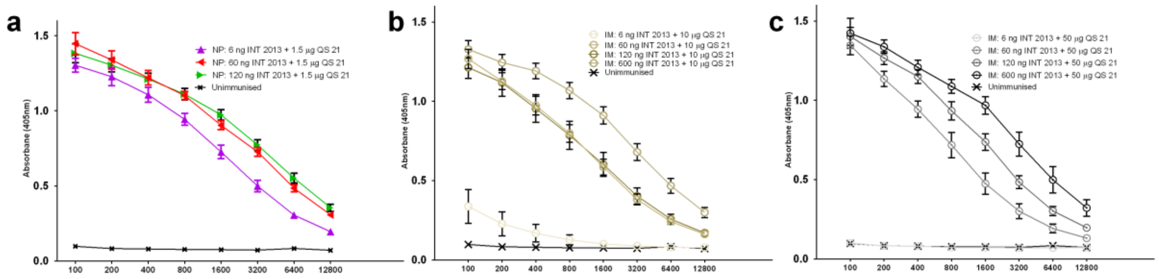

Supplementary Figure 3: Total serum antigen specific IgG response comparing QS-21 dose 21 days post immunisation at 1.5 µg delivered by Nanopatch, or 10 µg or 50 µg delivered by the needle and syringe intramuscular (IM) route. Nanopatch groups were represented by▲ (6 ng ▲, 60 ng ▶ and 120 ng ◀ of influenza antigen) ; IM groups were represented by ● (10 µg ○ or 50 µg of QS-21⊙)

with different dose of influenza antigen (6 ng, 60 ng, 120 ng and 600 ng). This figure (dilution curves) corresponds to Figure 4a (i), (ii) and (iii) (Endpoint titre).

a

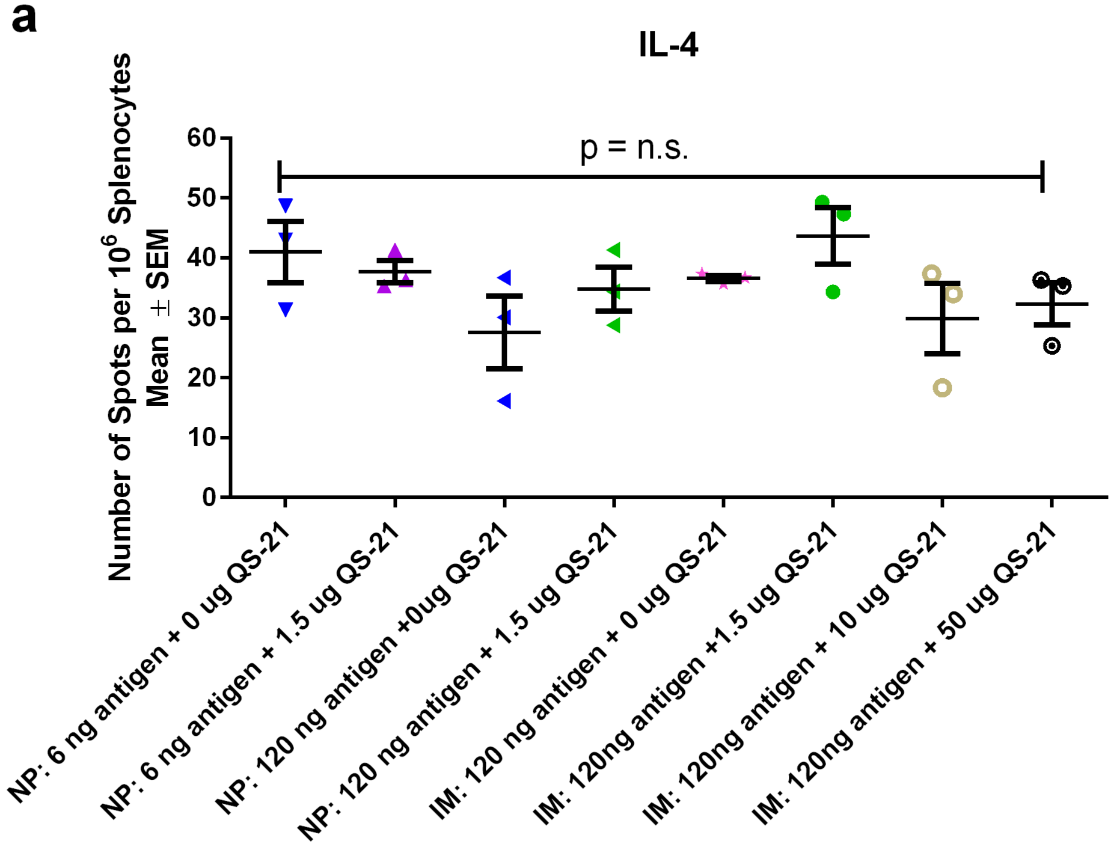

IL-4
Number of Spots per 10^6 Splenocytes Mean ± SEM
60
50
40
30
20
10
0
p = n.s.
NP: 6 ng antigen + 0 ug QS-21
NP: 6 ng antigen + 1.5 ug QS-21
NP: 120 ng antigen +0ug QS-21
NP: 120 ng antigen + 1.5 ug QS-21
IM: 120 ng antigen + 0 ug QS-21
IM: 120ng antigen +1.5 ug QS-21
IM: 120ng antigen + 10 ug QS-21
IM: 120ng antigen + 50 ug QS-21

b

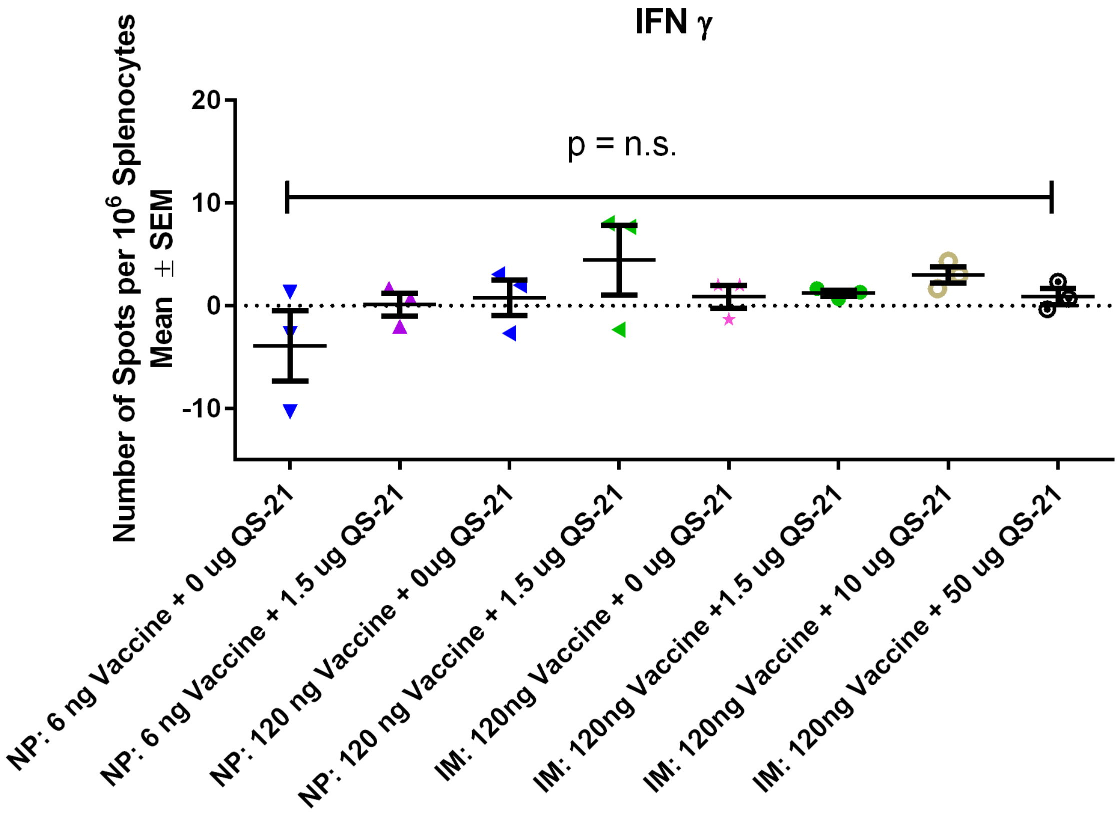

IFN γ
Number of Spots per 10^6 Splenocytes Mean ± SEM
20
10
0
-10
p = n.s.
NP: 6 ng Vaccine + 0 ug QS-21
NP: 6 ng Vaccine + 1.5 ug QS-21
NP: 120 ng Vaccine + 0ug QS-21
NP: 120 ng Vaccine + 1.5 ug QS-21
IM: 120ng Vaccine + 0 ug QS-21
IM: 120ng Vaccine +1.5 ug QS-21
IM: 120ng Vaccine + 10 ug QS-21
IM: 120ng Vaccine + 50 ug QS-21

Supplementary Figure 4: Antigen specific a) IL-4 and b) IFNγ ELISPOT induced by co-administering 6 ng or 120 ng of influenza vaccine with or without 1.5 µg of QS-21 by the Nanopatch, or co-administering various dose (1.5 µg, 10 µg or 50 µg) of QS-21 with 120 ng of influenza antigen by needle and syringe intramuscular (IM) route. Nanopatch groups were represented by ▲ unadjuvanted ▼ and QS-21▲) and IM groups were represented by ★,●,○ and ⊙ (unadjuvanted ★ and of QS-21● 1.5 µg, ○ 10 µg or ⊙ 50 µg). The number of spots was counted, averaged by 3 technical replicates and subtracted by the background (no peptide) wells. ELISPOT data represent the Mean ± SEM per million spleen cells, statistical significance is when $p<0.05$, one experiment of n=3 C57BL/6 mice per group. Statistical for endpoint titres were performed with one-way ANOVA. Equivalent of antigen specific IL-4 producing cells are induced by all the doses of NP and IM groups, which supported the earlier data. However, IFNγ ELISPOT were producing background related data (sub-zero points), which is highly possible to be due to the lack of specific peptide during re-stimulation. The reason for this limitation was identified by Zhong et al[47], influenza virus (PR8) contained major $CD8^+$ T cells epitopes were located in nucleoprotein and polymerase acidic protein, which is not in the antigen (purified HA protein from A/California, A/Victoria and B/Wisconsin) used in this study. The presence of $CD8^+$ T cells epitopes in HA protein were found to respond at a very low rate[47] and that different influenza strains may have different antigen presentation kinetics. Alternative ways developed by Ingulli et al[48], used ovalbumin-expressing influenza virus to infect their mice models and $CD8^+$ T cells epitope from ovalbumin (SIINFEKL) for re-stimulation to detect influenza specific $CD8^+$ T cell responses, which is a technique not available to our group.
